# Three-dimensional full-field X-ray orientation microscopy

**DOI:** 10.1038/srep20618

**Published:** 2016-02-12

**Authors:** Nicola Viganò, Alexandre Tanguy, Simon Hallais, Alexandre Dimanov, Michel Bornert, Kees Joost Batenburg, Wolfgang Ludwig

**Affiliations:** 1 MATEIS, INSA Lyon, CNRS (UMR5510), Univ. Lyon, F-69621 Lyon, France; 2 ESRF, The European Synchrotron, F-38043 Grenoble, France; 3 University of Antwerp, iMinds-Vision Lab, B-2610 Antwerp, Belgium; 4 LMS, Ecole Polytechnique, CNRS (UMR7649), Université Paris-Saclay, 91128 Palaiseau, France; 5 Univ. Paris-Est, Laboratoire Navier, ENPC, CNRS UMR8205, IFSTTAR, F-77455 Marne-la-Vallée, France; 6 Centrum Wiskunde & Informatica, Amsterdam, 1098 XG Amsterdam, The Netherlands; 7 University of Leiden, Mathematical Institute, 2300 RA Leiden, The Netherlands

## Abstract

A previously introduced mathematical framework for full-field X-ray orientation microscopy is for the first time applied to experimental near-field diffraction data acquired from a polycrystalline sample. Grain by grain tomographic reconstructions using convex optimization and prior knowledge are carried out in a six-dimensional representation of position-orientation space, used for modelling the inverse problem of X-ray orientation imaging. From the 6D reconstruction output we derive 3D orientation maps, which are then assembled into a common sample volume. The obtained 3D orientation map is compared to an EBSD surface map and local misorientations, as well as remaining discrepancies in grain boundary positions are quantified. The new approach replaces the single orientation reconstruction scheme behind X-ray diffraction contrast tomography and extends the applicability of this diffraction imaging technique to material micro-structures exhibiting sub-grains and/or intra-granular orientation spreads of up to a few degrees. As demonstrated on textured sub-regions of the sample, the new framework can be extended to operate on experimental raw data, thereby bypassing the concept of orientation indexation based on diffraction spot peak positions. This new method enables fast, three-dimensional characterization with isotropic spatial resolution, suitable for time-lapse observations of grain microstructures evolving as a function of applied strain or temperature.

Their ability to map crystallographic phase and orientation in three dimensions make X-ray diffraction imaging techniques a highly complementary tool to established 2D electron microscopy techniques like electron backscatter diffraction (EBSD).

In this article we present first experimental results obtained with a new six-dimensional data analysis framework^1^, applicable to monochromatic beam, near-field X-ray diffraction data, aiming at the reconstruction of spatially resolved, three-dimensional orientation maps. As opposed to 3D X-Ray Diffraction (3DXRD) variants based on pencil beam (1D) or line beam (2D) illumination which involve 3D or 2D scanning procedures limiting the ultimate acquisition speed, the full-field variant described in this paper requires only one rotational scan and can be executed on standard X-ray tomography instruments, offering simultaneous X-ray absorption and phase contrast inspection of the illuminated sample volume.

Depending on the experiment settings and material characteristics, the reconstruction task related to X-ray orientation microscopy can be sub-divided into one of the following three categories: (1) negligible intra-granular orientation spreads (2) presence of intra-granular orientation distributions which can be described as a 3D vector field and (3) presence of intra-granular orientation distributions which require a description as 3D orientation distributions per sampled volume element^2^.

In the first case the task of 3D grain shape reconstruction reduces to the classical problem of image reconstruction from parallel projections and can be solved using algorithms developed in the field of medical imaging^2^,^3^,^4^,^5^,^6^,^7^. Common to this class of micro-structure reconstruction techniques is a two step process based on poly-crystal indexing followed by grain by grain reconstruction. In the indexing step grain orientations and positions are identified based on a systematic search through the list of scattering vectors derived from the measured diffraction spot peak positions. In the second step, a projection stack and the corresponding projection geometry are constructed for each of the grains, which are reconstructed individually and assembled into the common sample volume. However, it has to be noted that the concept of indexing grain orientations from diffraction spot peak positions applies to materials and acquisition conditions giving rise to limited diffraction spot overlap on the detector. This concept is known to gradually fail with increasing complexity of the micro-structure and macroscopic plastic deformation (giving rise to increasing values of intragranular orientation spread and lattice strain) and eventually results in non-spacefilling grain maps (see Supplemental Information for a more detailed discussion of the interplay and the typical values of material and acquisition parameters determining the applicability of polycrystal indexing approaches).

Alternative strategies for X-ray orientation microscopy replace the above-mentioned two step process with a forward modeling approach performing a voxel-wise optimization in order to maximize the overlap between experimental data and the current description of micro-structure. These strategies are well suited for the second class of reconstruction task (i.e. description of the material micro-structure in terms of a 3D orientation field) and have proven to be applicable to samples which have undergone significant plastic deformation^8^. Prominent examples are the algorithms entitled ‘Grain Sweeper’^9^,^10^ and IceNine^11^,^12^, established in the frame of of the High Energy Diffraction Microscopy (HEDM) project at the Advanced Photon Source. Both algorithms share the concept of local (voxelwise) optimization and operate on a binarized version of the diffraction data.

In this article we introduce a framework which deals with the third, and most complicated case, accounting for a 3D orientation distribution for each sampled volume element. The formulation of the reconstruction task in six-dimensional position-orientation space was first proposed in earlier work by Poulsen^13^, but an actual implementation became only possible recently, thanks to progress in computing hardware and emergence of new concepts and algorithms in the field of mathematical optimization. Introducing a regular sampling over the sub-volume of 3D orientation space occupied by a grain, we have shown that the inverse and underdetermined problem of X-ray orientation microscopy can be solved with the help of first-order, convex optimization algorithms, using physical meaningful regularization terms^1^.

Whereas this previous work was based on synthetic diffraction data we present here a first application to experimental data acquired from a polycrystalline halite (rock-salt) sample containing more than 300 indexed grain orientations in the illuminated sample volume. We demonstrate that the quality and applicability of fast, full-field acquisition techniques can be significantly enhanced when switching to such a six-dimensional formulation of the reconstruction problem.

Three different types of grain maps, all reconstructed from the same set of experimental data are presented and compared to each other in this work. The first two maps are based on the two step process of indexing grain orientations, followed by algebraic reconstruction using the conventional 3D (single orientation) reconstruction algorithm^6^,^7^ and the new six-dimensional framework^1^, respectively. The material under study presents a pronounced grain sub-structure and some of these sub-grains could not be identified using conventional indexing schemes based on diffraction spot center of mass positions. We then present an extension of the six-dimensional approach, operating on experimental raw data and circumventing the steps of diffraction spot segmentation and orientation indexing. Seeded with approximate information concerning the real space and orientation space sub-volumes of the region to be reconstructed, this extended approach correctly identifies the missing sub-grain orientations and results in a space-filling grain map.

For visualization and evaluation of the reconstruction results we used the reduced, 3D vector field representation of the orientation field, derived from the 6D reconstruction output. The orientation of a real space voxel in this representation is calculated as the center of mass of the local orientation distribution associated to this voxel. The resulting 3D orientation maps have been compared to EBSD measurements acquired on one of the lateral sample surfaces and local disorientation and discrepancies in the grain boundary positions have been analysed.

## Method

In a monochromatic beam, near-field diffraction experiment the sample is positioned on a rotation stage, and as it rotates by angle *ω*, it gives rise to diffracted beams each time the Bragg condition is met for one of the grains. A limited number of the diffraction spots will intersect the high resolution imaging detector positioned a few millimeters downstream of the sample. Each of the sub-orientations present in a grain is associated to a slightly different projection geometry and the diffraction signal associated to a given Bragg reflection is observed as a three-dimensional diffraction “blob” volume, parametrized by two spatial coordinates *u* and *v* (detector pixel coordinates) and a rotation angle *ω* (image number).

Whereas in the previous implementations of DCT the reconstruction units were grains, described by an average orientation and a corresponding projection geometry applied to the integrated diffraction spots (2D), we now introduce an explicit, discrete sampling of the local orientation space centered around the grain average orientation and exploit the additional information encoded in the intensity variation of the 3D diffraction blob volumes as a function of the rotation angle. We neglect the possible (in metals typically ≤1%) elastic distortion of the crystal unit cell and introduce a 6-dimensional reconstruction space 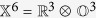 obtained by the outer product of cartesian real-space and 3-dimensional orientation space.

Assuming kinematic diffraction and neglecting photoelectric absorption and extinction effects, the process of diffraction image formation (forward projection) can be formulated as a linear operator:


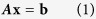


where **x** is a vector containing *NP* elements, representing the scalar “scattering power” for each of the sampled volume elements in the six-dimensional position-orientation space (*NP* = *n*^3^ × *p*^3^ for the case of a regular sampling over cube-shaped sub-volumes with grid length *n* and *p* in position and orientation space, respectively). Each line of the projection matrix ***A***_*S*×*NP*_ contains the contribution of the 6D volume elements to a given detector pixel and the vector **b**_*S*_ holds a list of measured pixel intensities, specified by their (*u*, *v*, *ω*) coordinates in the 3D diffraction image stack. *S* corresponds to the total number of detector pixels in the 3D image stack reached by the *M* projections (*hkl* reflections) of the grain volume(s).

If the elements of **x** are arranged as a succession of 3D real space volumes, each representing one of the sampled orientations, and ***A*** as an array of *M* × *P* projection matrices, each one describing the projection geometry for one of the *M hkl* reflections intercepted by the detector, the vector **b** will be composed of *M* blocks corresponding to contiguous 3D subvolumes (i.e. the aforementioned diffraction blobs), spread throughout the entire stack of detector images:


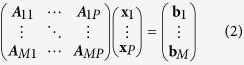


(Removing lines of **b**_*i*_ with elements corresponding to *ω* positions not reached by current orientation *j*, the resulting sub-matrices 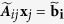 correspond to parallel beam projections of single orientation volumes onto 2D diffraction spot images 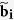, as used in previous 3D implementations of DCT^6^).

### Formulation as a 6D optimization problem and underlying assumptions

While equation (2) is a concise formulation of the diffraction image formation (forward projection), it is not suited for the actual reconstruction. Whereas in the case of 3D reconstructions based on single (grain average) orientation one had to solve for one unknown (local scattering power) per real space volume element, we now have to solve for a much larger number of unknowns (i.e the scalar scattering power for each of the discretized orientations (~*p*^3^ - typically several hundreds up to a few thousands of orientations per real space voxel). At the same time the number of measurements typically only increases by a factor of *p*^1^. Moreover measurements from a polycrystalline sample are affected by diffraction spot overlaps from other grains and various sources of noise.

As a consequence, equation (2) is an ill-posed, heavily under-determined problem and has to be re-written as a minimisation problem, in which case additional terms enforcing physical meaningful constraints and prior knowledge about the solution can be incorporated in its optimization functional.

While in previous work on synthetic diffraction data^1^ we chose to use an *l*_1_ minimization over the full six-dimensional space, because of the *a priori* knowledge that the phantom was based on a (sparse) 3D vector field representation of the grain volume, in the current work we used a slightly different formulation (eqs. 3 and 4) which promotes positive solutions minimising spatial variations of the integrated scattering power assigned to the real space voxels, as to be expected from a purely kinematic diffraction model.


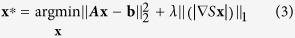



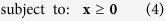


where *S* is the operator that sums all the orientation components for each real-space voxel, and the *l*_1_-norm over the absolute value of the gradient is the total variation operator^14^,^15^.

This formulation is well adapted to the reconstruction of materials showing a pronounced sub-grain structure, since cross-talk between the sampled orientations tends to assign intensity to the grain boundary regions and results in sharper boundaries and better homogeneity of the reconstructions compared to the previous *l*_1_-norm formulation.

Like in the previous work^1^, a specific class of algorithms called Chambolle-Pock^16^ is used for the minimization of the functional in equation 3. As shown in the supplementary material this functional can be rewritten in a different form, leading to a very similar algorithm, but now with a fixed weight *λ* = 1, more suitable for practical work on large dataset containing a large number of individual reconstruction problems.

For more details on the calculation of the projection matrix, on the 6-dimensional space and on the optimization algorithm, we refer to the supplementary material and^1^,^2^,^17^.

## Results

**Figure 1:**
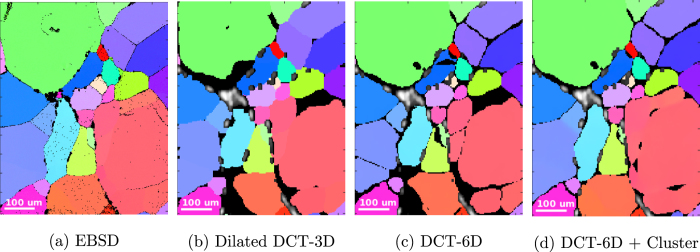
Comparison of EBSD surface mapping with the different reconstruction approaches for full-field X-ray orientation microscopy discussed in this work.

A parallelepiped sample with 0.9 × 0.9 × 2.2 mm dimension was prepared from a two-phase material consisting of large (100–400 *μ*m) NaCl grains and a fine dispersion of micrometer sized Cu particles. (The fine dispersion of Cu serves as a contrast agent for digital volume correlation and will be used for determination of the 3D displacement fields introduced by incremental compressive loading of the specimen, foreseen in a follow-up experiment.) Halite is a visco-plastic geomaterial deforming under the action of grain boundary sliding and dislocation mediated plasticity^18^,^19^. Here we report on the characterization of the 3D orientation field prior to plastic deformation of the specimen. Closer inspection of the EBSD surface mapping (Fig. 1a) reveals that part of the bigger grains consist of smaller sub-grains with typical dimensions of order of 100 *μ*m and which are delineated by small angle boundaries with a few degrees of misorientation. A 560 *μ*m high sub-volume of the specimen containing more than 300 grains was scanned on a conventional X-ray imaging setup available at the bending magnet beamline BM05 of the European Synchrotron. After the synchrotron experiment one of the lateral sample surfaces was characterized by electron backscatter diffraction for comparison and cross-validation of the X-ray orientation maps calculated from the X-ray diffraction data.

More details on the sample preparation and experiment conditions used for the DCT and EBSD characterization are given in the supplementary material.

### DCT reconstructions

Three different types of grain reconstructions were performed in order to illustrate the improvements of the new reconstruction framework compared to the previous (single orientation) approach: (1) a standard 3D DCT reconstruction of the grains that have been identified in the polycrystal indexing step. Integrated 2D diffraction spot images were used for the reconstruction and the resulting 3D grain map underwent a two voxels dilation step as described in^6^ (2) a 6D DCT reconstruction of the same set of grains, but now reconstructed from the 3D diffraction blob volumes using a variant to of the recently introduced 6-dimensional algorithm^1^ and (3) an extension of the latter where sub-regions of the 3D orientation map corresponding to some extended regions in real space and orientation space have been reconstructed from unsegmented experimental raw data.

As mentioned earlier, the 6D reconstruction output has been reduced to the conventional 3D vector map representation, commonly used in orientation imaging microscopy. In all three cases the final shape of the 3D grain volumes is determined by an automated thresholding operation at the time of assembling the individual grain reconstructions into the common sample volume. Note that no additional dilational postprocessing was applied to the grain orientation maps obtained by the six-dimensional reconstruction approaches.

### Grainmaps comparison

Figure 1 shows a comparison of the EBSD map acquired close to one of the sample surfaces with the corresponding section through the reconstructed grain volume obtained with the three approaches described in the previous section.

The grain maps are color coded according to the inverse pole figure mapping along the surface normal and overlaid on the X-ray attenuation map (as explained in Supplementary Information the DCT volume and orientation matrices have been rotated by about 2.4 degrees in order to coincide with the reference frame of the back-scatter electron image. The IPF colour key is provided in Figure S1.) The absorption image has been reconstructed from the simultaneously acquired X-ray projection images and it is intrincically aligned with the DCT grain map. Due to their higher attenuation coefficent the Cu particles show up as bright particles, decorating part of the high angle grain boundaries in the NaCl matrix material.

The construction of the depicted X-ray orientation maps involves the steps of segmentation and assembly of individually reconstructed grain volumes into the common sample volume. This process may lead to non-space-filling orientation maps in case of grain orientations not been identified in the previous indexing step. Inspection of the EBSD map indicates that the “holes” in proximity of the orange and rose grains at the bottom and right side of the depicted grain maps Fig. (1b,c) correspond to sub-grains separated by low-angle boundaries with less than 3° misorientation. Since the connectivity of diffraction blobs originating from grains with a pronounced sub-structure changes as a function of the (*hkl*) reflection, the center of mass and shape based indexing procedures outlined in^6^,^7^ may fail to identify part of the existing sub-grains in this situation. However, as long as one of the sub-grains has been identified a straightforward extension of the six-dimensional reconstruction approach discussed in the next section can be used to find the others and eventually results in a space-filling grain map (Fig. 1d) with a much reduced number of ambiguously or unassigned voxels.

As the attenuation map is simultaneously measured with the 3D orientation map, with the same detector, in the same reference system, it is possible to complement the grain map with the information about the position of the Cu particles. This type of information is also interesting because the Cu particles are only observed at the grain boundary positions, and their presence is both a direct confirmation of a correct indexing and a measure of the spatial accuracy of the reconstructed grain maps.

### Reconstruction of sub-grain clusters and intra-granular orientation

Having identified one of the sub-grain orientations in a textured and/or badly reconstructed region of the sample a straight forward extension of the current framework allows to identify and reconstruct neighboring sub-grains without the need to explicitly identify and isolate the corresponding diffraction blobs on the detector. It is enough to extend the real-space bounding box to include the missing region, and to (iteratively) extend the orientation-space bounding box until the missing sub-grains are fully included. A new stack of diffraction blobs (*difstack* as defined in^1^), that would include the region on the detector covered by the forward projection of the extended real-space and orientation-space volumes is assembled and then reconstructed with the very same 6D algorithm used for the indexed grains.

**Figure 2:**
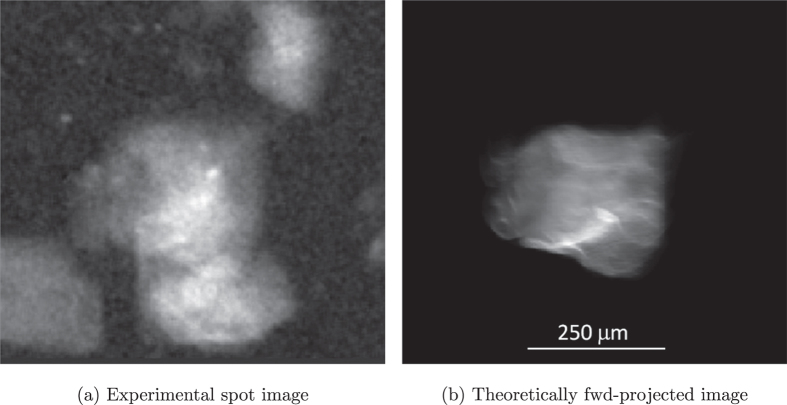
Comparison of the experimental images for an [2 2 2] reflection at *θ* = 6.21 *degrees*, *η* = 112  *degrees*, with a Δ*ω* = 6.7 *degrees* (67 images), with the same forward-projected spot from the result of the reconstruction.

**Figure 3:**
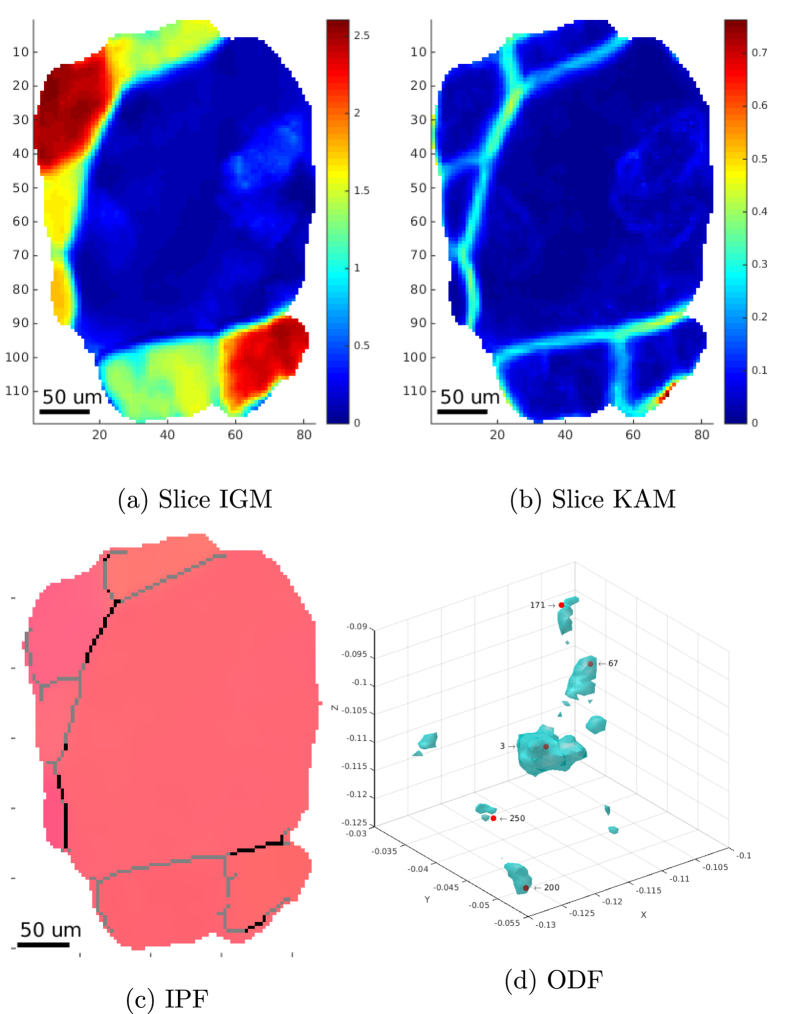
Reconstruction of a grain cluster using the extended 6D approach. (**a**–**c**) same slice through the 3D reconstruction showing: (**a**) Intra-granular Misorientation, (**b**) Kernel Average Misorientation (**c**) inverse pole figure colour coding scheme revealing the presence of sub-grains and small angle boundaries from skeletonization of (**a**) (gray: ≥0.5°, black: ≥2°), (**d**) iso-surface of the orientation sub-space reconstructed for the clustered region. Red points indicate sub-grain orientations which had been successfully identified using conventional indexing procedures, along with their corresponding grain ID.

There is, however, one important difference: unlike the difstack of indexed grains which is constructed from a subset of pre-selected and segmented diffraction blob volumes, the difstack corresponding to a clustered region is directly assembled from the background corrected experimental raw images. This in turn may lead to considerable degree of overlap between the reflections of the grain under analysis and spurious reflections from other grains, as depicted in Fig. (2a). In fact, these spurious overlaps are mathematically inconsistent to each other, from one blob to another one, and the reconstruction algorithm is able to greatly reduce their impact on the final reconstruction. Indeed, Fig. (2b) shows what the forward projection of the segmented reconstruction volume reproduces the diffraction blob corresponding to the grain under investigation (the grain cluster depicted in Fig. 3c), and the overlaps, affecting about 50% of the projections, are completely filtered out. It should be noted that even with indexed spots overlaps may occur, so this tolerance is important.

Figure (3a) is a zoom on the clustered region in the right mid-bottom part of Fig. (1d). It shows that the intra-granular misorientation is characterized by a maximum value of 2.5 *degree*s misorientation from the chosen reference orientation and illustrates the local orientation characterization capability of the new six-dimensional reconstruction framework. Each of the real space voxels carries a local orientation determined from the weighted average of the intensity assigned to the 3D orientation space voxels related to this specific position.

The calculation of the kernel average misorientation in Fig. (3b) clearly reveals the presence of small-angle grain boundaries in this region. Note that these boundaries are not sharp, which can be partly attributed to the fact that the orientation-space resolution for this calculation was limited to a grid of 11 × 11 × 11 orientations over a bounding box of 3.1° × 2.5° × 3.9°, because of memory constrains.

Interesting insight about the structure of the solution is also gained from inspection of the local 3D orientation space associated to this sub-structured region of the sample volume. Figure (3d) confirms that the algorithm was able to identify a few new sub-grain orientations in addition to the five indexed sub-grain orientations detected by the conventional indexing procedure. The application of this extended reconstruction framework to other regions which were incompletely reconstructed in Fig. (1c) results in a significant improvement of the reconstruction which now is close to space-filling (Fig. 1d).

**Figure 4:**
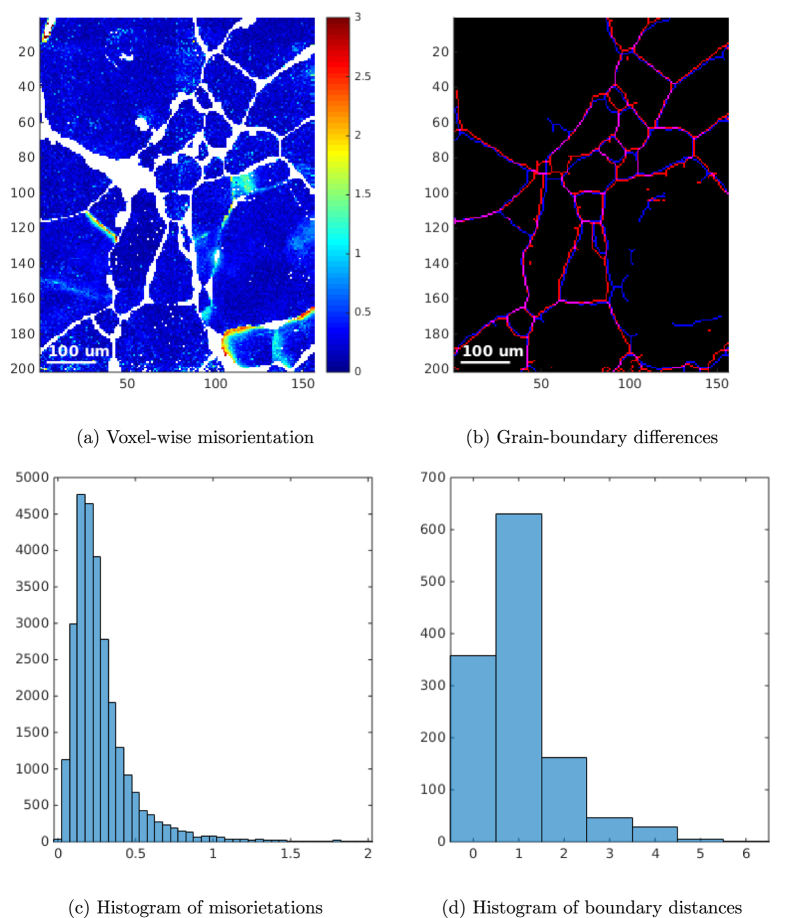
Quantitative comparison between EBSD map and the selected surface slice in the 6D-DCT volume, where we can find: (**a**) voxel-wise distance in degrees between the orientations found by EBSD and 6D-DCT, (**b**) Overlay of grain-boundaries from EBSD (red) and 6D-DCT (yellow), (**c**) Histogram of misorientations in (**a**,**d**) Histogram of pixel distances in (**b**).

Finally, Fig. (4), shows a quantitative comparison between the EBSD map in Fig. (1a) and the 6D-DCT map in Fig. (1d). More precisely, Fig. (4a) shows the voxel-by-voxel misorientation between the two maps. Regions corresponding to Cu particles and voxels close the grain boundaries, assigned to different grains are shown in white and have been excluded from this analysis. The histogram of the misorientation distribution Fig. (4c) shows a pronounced peak at 0.2 degrees and some low intensity tails up about 1 degree misorientation.

In Fig. (4b) instead, an overlay of the grain boundaries computed with the two techniques is presented. The local distance between the two maps has been determined by intersecting the (binary) DCT boundary map with the distance transform calculated from the EBSD boundary map. The corresponding histogram with an average distance of 1.06 voxels (corresponding to 3.7 *μ*m) is presented in Fig. (4d).

## Discussion

With the transition from a 3D to a 6D reconstruction framework, the previous restriction of diffraction contrast tomography to materials with negligile values of intragranular orientation spread disappears. By assiging an average orientation to each of the reconstructed voxels, the output becomes identical to well established 2D and 3D grain mapping techniques based on scanning electron or X-ray diffraction techniques. However, an important difference remains: unlike scanning techniques or reconstruction schemes based on voxel by voxel optimization, the global optimization approach as presented in this article was seeded with some approximate information concerning grain position and orientation, obtained from a previous polycrystal indexing step. This implies a remaining restriction to moderately deformed microstructures (typically ≤5% plastic deformation), which can be still be described as a collection of grains, representing 3D crystal domains with limited orientation spread and well-defined boundaries (see Supplemental Information for a discussion of the requirements for successful orientation indexation from diffraction spot peak positions).

As expected from a previous feasibility study on synthetic diffraction data, the transition from the previous single-orientation (3D) to a 6D reconstruction framework results in a visible improvement in the accuracy of the grain shape reconstructions. The extended model can account for the non-parallel projection geometry of deformed grains and the iterative reconstruction process produces consistent grain shapes which can be assembled into the 3D sample volume with much reduced tendency for overlaps (i.e. voxels simulateneously claimed by adjacent grains) and reduced gaps between grains, corresponding to unassigned voxels. Indeed figures 1 and 4 confirm that the grain boundaries obtained with the 6D-DCT approach (using the “grain cluster” generalization for the clustered regions and no dilational postprocessing of the grain map) are in good agreement with the EBSD measurements. The accuracy of the grain boundary positions determined with the 6D method appears to approch the voxel size of the reconstruction (3.75 *μ*m in this study). It can be expected that values down to 1 *μ*m resolution can be reached when working with high resolution configurations of the detector system, as typically employed in studies of materials with grain sizes in the range of 10–50 *μ*m.

For what concerns the reconstruction of local orientations, Fig. (4a,c) suggest that also in this case the algorithm is able to retrieve an average orientation for each voxel that is close (i.e. within the accuracy of the EBSD measurement) to the results obtained from EBSD. The full width at half maximum of the misorientation distribution (0.25 degree) is comparable to the expected accuracy of the EBSD measurement. As explained in the Supplementary Information, the overall shift of about 0.2 degree in misorientation can be explained by remaining uncertainties in the alignment of the real-space and orientation space reference systems of both measurement modalities.

We furthermore recall that the results presented in Fig. (1c,d) are a projection of the full six-dimensional reconstruction output into the 3D vector field representation that describes the local average orientations in each voxel. This means that by using this representation we have lost the information concerning the possible presence of sub-voxel orientation domains. Further tests are required in order to evaluate the accuracy of the local (per voxel) 3D orientation distribution output by our optimization routine. Note that the 2D EBSD measurements presented in this study are not sufficient for giving a ground truth for the six-dimensional reconstruction, since the interaction volume and information depth (of order of 100 nm) of EBSD is much smaller than the voxel size (3.75 *μm*) used in this study. Three-dimensional, high angular resolution 3D EBSD on dual-beam FIB-SEM instruments^20^ or Differential Aperture X-ray Microscopy (DAXM^21^) measuements shall be considered for this task.

The current model is based on an idealized (purely kinematic) description of the diffraction process and calculations are performed on diffraction blob volumes which have been renormalized to constant intensity, which is a pragmatic but rather crude way to account for intensity variations due to differences in structure and Lorenz factors, spatial and temporal inhomogeneity of the incoming beam profile and attenuation of the incoming and diffracted beam due to photoelectric absorption and extinction effects. The model could in principle be extended to account for these effects: structure and Lorenz factors can be calculated and the incoming beam profile is periodically updated by taking images without the object during the tomographic acquisition procedure. Moreover, the 3D attenuation coefficient distribution is reconstructed from the transmitted beam and after a first 3D reconstruction one could in principle check if grains on the trajectory of the incoming and diffracted beam simultaneously fulfill the diffraction condition, giving rise to local intensity variations.

An obvious extension of the approach applied to the sub-grain clusters would consist in enlarging the sampling of the orientation space to the entire fundamental zone. Covering the fundamental zone for high symmetry space groups like the cubic with an orientation resolution of 0.2 degrees would require of order of 10^8^ degrees of freedom. With currently available computing hardware featuring up to 512 GB of memory, the model could possibly handle high-resolution diffraction data acquired in slice beam illumination mode.

Another promising development route would consist in simultaneous acquisition of full field (extended beam) diffraction data on 3D detector systems featuring two or more screens with different pixel size and positioned at different distances. The reduced number of spatial degrees of freedom on the outermost low spatial resolution (diffraction) detector would allow the identification of orientations and approximate positions^9^,^22^ which in turn can be reconstructed at higher spatial resolution from the near-field diffraction data, using the extended approach presented in the current paper.

The design and implementation of the reconstruction algorithm presented here is also independent from the type of tomographic projector used and could be easily modularized to allow for polychromatic projectors^23^ to be used in both polychromatic X-ray or neutron imaging applications^24^. The advantage of using such framework over the previous approaches would be once again in the possibility to explicitly take the distortion of diffraction images due to the local variations of the diffraction angles into account. Due to additional variation of the Bragg angle, these distortions are known to be more severe in polychromatic imaging^24^ and severely restrict the choice of materials which can be analysed in the single orientation framework.

As a final remark, it should be stated that the current work is based on a 6-dimensional kinematic diffraction model which does not take into account the possible distortion of the unit cell as result of elastic strains, which would require additional six degrees of freedom. Due to the exponential growth of memory requirements with the number of sampled dimensions the discrete sampling approach used in this work will not be an appropriate framework for addressing the full 12-dimensional problem.

## Conclusions

We have demonstrated the feasibility of three-dimensional full-field X-ray orientation microscopy from extended beam near-field X-ray diffraction data. A new six-dimensional reconstruction framework yields spatially resolved 3D attenuation and orientation maps, substituting and outperforming the previously introduced three-dimensional (single orientation) reconstruction algorithm behind X-ray diffraction contrast tomography.

The results obtained on a two phase material made from NaCl and containing a fine dispersion of Cu particles have been cross-validated against EBSD measurements on the sample surface and indicate that our approach yields an orientation resolution comparable to EBSD and a 3D spatial resolution consistent with the pixel size of high resolution X-ray imaging detectors.

As demonstrated on a material displaying a pronounced grain sub-structure, the introduced six-dimensional frame can be extended to operate on non-segmented raw data corresponding to sub-volumes of the six-dimensional position-orientation space. This finding indicates possible future extensions of the framework, such as replacing the two-step process of orientation indexing and reconstruction by a global optimization procedure by including extended regions of orientation space into the reconstruction process.

Finally, as a full-field approach, compatible with simulateneous absorption and phase contrast imaging, the proposed methodology is currently about one order of magnitude faster than competing techniques based on slice or pencil beam illumination. This specific feature opens interesting new possibilities for time-lapse observations of processes like plastic deformation, coarsening or phase transformations in bulk polycrystalline structural materials.

## Additional Information

**How to cite this article**: Viganò, N. *et al.* Three-dimensional full-field X-ray orientation microscopy. *Sci. Rep.* **6**, 20618; doi: 10.1038/srep20618 (2016).

## Supplementary Material

Supplementary Information

## References

[b1] Viganò N., Ludwig W. & Batenburg K. J. Reconstruction of local orientation in grains using a discrete representation of orientation space. J. Appl. Crystallogr. 47, 1826–1840 (2014).

[b2] Poulsen H. F. A six-dimensional approach to microtexture analysis. Philos. Mag. 83, 2761–2778 (2003).

[b3] Gordon R., Bender R. & Herman G. T. Algebraic Reconstruction Techniques (ART) for three-dimensional electron microscopy and X-ray photography. J. Theor. Biol. 29, 471–481 (1970). 5492997 10.1016/0022-5193(70)90109-8

[b4] Gregor J. & Benson T. Computational analysis and improvement of SIRT. IEEE Trans. Med. Imaging 27, 918–24 (2008). 18599397 10.1109/TMI.2008.923696

[b5] Markussen T. *et al.* An algebraic algorithm for generation of three-dimensional grain maps based on diffraction with a wide beam of hard X-rays. J. Appl. Crystallogr. 37, 96–102 (2004).

[b6] Ludwig W. *et al.* Three-dimensional grain mapping by x-ray diffraction contrast tomography and the use of Friedel pairs in diffraction data analysis. Rev. Sci. Instrum. 80, 033905 (2009). 19334932 10.1063/1.3100200

[b7] Reischig P. *et al.* Advances in X-ray diffraction contrast tomography: flexibility in the setup geometry and application to multiphase materials. J. Appl. Crystallogr. 46, 297–311 (2013).

[b8] Li S. F. *et al.* Three-dimensional plastic response in polycrystalline copper *via* near-field high-energy x-ray diffraction microscopy. J. Appl. Crystallogr. 45, 1098–1108 (2012).

[b9] Schmidt S. *et al.* Direct observation of 3-D grain growth in Al-0.1% Mn. Scr. Mater. 59, 491–494 (2008).

[b10] Borthwick V. E., Schmidt S., Piazolo S. & Gundlach C. Quantification of mineral behavior in four dimensions: Grain boundary and substructure dynamics in salt. Geochem. Geophys. Geosyst. 13 (2012).

[b11] Suter R. M., Hennessy D., Xiao C. & Lienert U. Forward modeling method for microstructure reconstruction using x-ray diffraction microscopy: Single-crystal verification. Rev. Sci. Instrum. 77, 123905 (2006).

[b12] Li S. F. & Suter R. M. Adaptive reconstruction method for three-dimensional orientation imaging. J. Appl. Crystallogr. 46, 512–524 (2013).

[b13] Poulsen H. F. & Fu X. Generation of grain maps by an algebraic reconstruction technique. J. Appl. Crystallogr. 36, 1062–1068 (2003).

[b14] Candes E. & Romberg J. l1-magic: Recovery of sparse signals via convex programming. *URL* http://users.ece.gatech.edu/justin/l1magic/downloads/l1magic.pdf (2005). (Date of access: 22/12/2015).

[b15] Sidky E. Y., Jørgensen J. H. & Pan X. Convex optimization problem prototyping for image reconstruction in computed tomography with the Chambolle-Pock algorithm. Phys. Med. Biol. 57, 3065–91 (2012). 22538474 10.1088/0031-9155/57/10/3065 PMC3370658

[b16] Chambolle A. & Pock T. A First-Order Primal-Dual Algorithm for Convex Problems with Applications to Imaging. J. Math. Imaging Vis. 40, 120–145 (2010).

[b17] Bernier J. V., Barton N. R., Lienert U. & Miller M. P. Far-field high-energy diffraction microscopy: a tool for intergranular orientation and strain analysis. J Strain Anal. Eng. 46, 527–547 (2011).

[b18] Skrotzki W. & Welch P. Development of texture and microstructure in extruded ionic polycrystalline aggregates. Tectonophysics 99, 47–61 (1983).

[b19] Yahya O., Aubertin M. & Julien M. A unified representation of the plasticity, creep and relaxation behavior of rocksalt. Int. J. Rock Mech. Min. 37, 787–800 (2000).

[b20] Wilkinson A. J. & Britton T. B. Strains, planes, and EBSD in materials science. Mater. Today 15, 366–376 (2012).

[b21] Larson B. C., Yang W., Ice G. E., Budai J. D. & Tischler J. Z. Three-dimensional X-ray structural microscopy with submicrometre resolution. Nature 415, 887–890 (2002). 11859363 10.1038/415887a

[b22] Kazantsev I. G., Schmidt S. & Poulsen H. F. A discrete spherical x-ray transform of orientation distribution functions using bounding cubes. Inverse Probl. 25, 15 (2009).

[b23] van Aarle W., Ludwig W., King A. & Penumadu D. An accurate projection model for diffraction image formation and inversion using a polychromatic cone beam. J Appl. Crystallogr. 48, 334–343 (2015).

[b24] King A., Reischig P., Adrien J., Peetermans S. & Ludwig W. Polychromatic diffraction contrast tomography. Mater. Charact. 97, 1–10 (2014).

