# Supplementary material for: Three-dimensional full-field X-ray orientation microscopy
Source: Sci Rep. 2016 Feb 12;6:20618. doi: 10.1038/srep20618 (PMC4751536; doi:10.1038/srep20618)
Supplement: Supplementary Information [file srep20618-s1.pdf]

Three-dimensional full-field X-ray orientation microscopy

Nicola Viganò, Alexandre Tanguy, Simon Hallais, Alexandre Dimanov
Michel Bornert Kees Joost Batenburg, Wolfgang Ludwig

Supplementary Information

EXPERIMENTAL DETAILS

X-ray diffraction contrast tomography data acquisition

X-ray diffraction contrast tomography measurements were performed at the bending magnet beamline BM05 of the European Synchrotron Radiation Facility (ESRF). The central part of a parallelepiped sample was illuminated by a monochromatic X-ray beam (35 keV; 1.5 (h) $\times$ 0.56 (v) mm cross-section), delivered by a Si 111 double crystal monochromator. The transmitted and diffracted beams were captured on a detector system positioned 15 mm downstream of the sample position. The detector system consisted of a back-illuminated Frelon CCD camera, coupled via visible light optics to a $100\,\mu$m thick transparent luminiscent screen made of LuAG [1] and provided an effective pixel size of $3.75\,\mu$m and a field of view of 7.7×7.7 mm. 3600 images with an exposure time of 8 sec were recorded during a $360°$ continuous rotation movement of the sample. With these settings the five innermost Debey-Scherrer rings were intercepted by the detector and on average 45 out of the 60 diffraction blobs could be unambiguously assigned to one of the 345 indexed grains in the illuminated sample volume.

EBSD acquisition

In order to compare the 6D-DCT reconstructions to a classical EBSD surface measure-

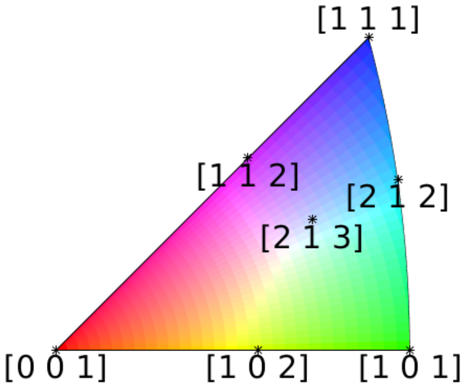

FIG. 1: IPF Color key used for the orientation coloring in figure (1).

ment, the surface of the sample was further prepared by broad argon ion beam sputtering, using the most recent ion-beam polishing system from Gatan [PECS-II], which combines cross sectioning and planar polishing capabilities. In order to prepare a large mm-sized area, we used the planar polishing technique, applying an acceleration voltage of $2\,kV$ and $5°$ beam incidence, for a duration of about 1 hour.

The EBSD data were acquired on an environmental Quanta 600 FEG-SEM instrument, operated at 20 Hz acquisition rate, an acceleration voltage of $20\,kV$, a working distance of 17.6 mm and a residual air pressure of 100 Pa in order to avoid surface charging. A grid of 5×4 partially overlapping EBSD maps, of $300 \times 270\,\mu$m size each, allowed for covering an entire lateral sample surface with a step size of 1.3 μm. The acquired diffraction patterns were analyzed using the AZtec HKL system from Oxford Instruments. The standard procedure with a minimum of 12 indexed Kikuchi lines allowed for 99.6% indexation success. A surface layer of approximately $20\,\mu$m in depth was removed in five successive ion-sputtering steps before a full correspondence between the micro-structures observed by EBSD and DCT was obtained.

IPF coloring

The coloring key used for figure (1) in the text of the article is reported in figure (1).

Registration of datasets and remaining sources of error

The two-dimensional EBSD mapping and the three-dimensional tomographic reconstruction have been carefully aligned with respect to each other, using an optimization routine based on the position of a set of Cu particles identified in both imaging modalities. Cu particles are visible in the 3D absorption image and in the backscatter electron image acquired in conjunction with the EBSD characterization. The optimization provides a best fit between the X-ray absorption map and the electron backsacttered electron image of the polished sample surface.

Nevertheless, the histograms in figure 4 show some remaining discrepancies. Whereas part of these discrepancies may be attributed to actual errors introduced by the reconstruction and segmentation procedure (due to uncertainties in the experiment geometry, non-idealities of the detector system and various other sources of noise), a series of other factors complicate a one to one comparison between both imaging modalities:

1. The voxel size of the DCT map (3.75 μm in our case) is significantly bigger than the typical interaction volume of EBSD measurements (of order of 100 nm). Even though the EBSD maps have been sampled with a smaller step size of $1.3 \mu m$, the volumes from which the average orientations in both maps are derived from are not identical.

2. The comparison is carried out between a not ideally flat surface (there is some remaining curvature and surface relief created by the ion beam polishing procedure) and a geometric plane inside a 3D volume.

3. There may be some remaining spatial distortion of the (stitched) EBSD map after the affine transformation (70 degree tilt) back into the coordinate system aligned with the electron beam direction.

4. The alignment of the orientation-space reference system between the two datasets does not perfectly coincide with alignment of the real-space reference systems: while the real-space alignment suggested a rotation of 2.6 *degree*s, the orientation-space alignment suggested a rotation of only 2.4 *degree*s. This is however is in-line with the observation of a shift in the peak of misorientation histogram shown in figure 4d (main text). We attribute this to a remaining curvature of the sample surface and

small errors in the calibration between the reference frames of the EBSD map and the back-scattered electron image.

5. The distance transform calculated in 2D can only provide a conservative estimate of the true distance in three-dimensions (boundaries cutting the observation plane at a small angle, can give rise to large errors).

SAMPLE PREPARATION

This work is part of a research project aiming at the characterization of plastic deformation mechanism in Halite (rock-salt, NaCl) by combined use of digital volume correlation (DVC) [2, 3] and 3D grain mapping techniques. Abandoned rock-salt mines are currently considered as natural reservoirs for compressed air energy storage power plants, and there are questions concerning creep deformation and damage modes of salt as a geo-material [4]. In order to enable the characterization of 3D displacement fields in the bulk of the polycrystalline microstructure as a function of applied plastic deformation a two-phase material providing internal (absorption) contrast was prepared for this study. Pure synthetic NaCl powder and a fine grained dispersion of Cu particles (3 volume %, average size 3 μm) were mechanically mixed at room temperature. A nearly dense cylindrical block of 7 cm in diameter, with about 3 % of porosity and widely spread NaCl grain size distribution (20 - 300 μm) was obtained after a first stage of hot isostatic pressing (HIP) at a temperature of 150°C and 100 MPa for 168 h.
In order i) to promote NaCl grain growth to a size compatible with DCT characterization of a mm-sized specimen, and ii) to reduce the residual porosity for the purpose of the mechanical testing, additional high temperature HIPing of cm-sized cylinders was performed at 700°C and 2.5 MPa over 24 hours. The latter procedure resulted in a homogeneous two-phase micro-structure, consisting of equiaxed NaCl grains with narrow size distribution (100 - 400 μm) and an average size of about 250 μm (as determined by the linear intercept method, considering grain boundaries with $\geq$ 10 degrees misorientation) and a residual porosity of less than 1 vol%. Interfacial dragging of the copper particles during NaCl grain growth did result in a micro-structure presenting individual NaCl grains and clusters of a few NaCl grains delineated by a more or less continuous 3D network of segregated Cu particles. A parallelepiped shaped sample was extracted from the center of the hot pressed material

using a low speed diamond saw, and was carefully polished down to grid 4000 SiC paper to final dimensions of $0.9 \times 0.9 \times 2.2$ mm.

Even if the material was tailored for this type of combined study, the micro-structure of the material shows very similar characteristics to the microstructures encountered in stuctural materials like metals and their alloys. In both cases the material can deform through dislocation mediated plasticity, and dislocations may organize in sub-structures leading to the formation of cells and small-angle grain boundaries.

SAMPLE REQUIREMENTS

Although the full-field diffraction approach presented in this work can deliver comparable output to other well established orientation imaging techniques, the higher convolution of the diffraction signal (illumination of the full 3D sample volume) imposes more stringent conditions on the type of microstructures which can be successfully analysed, as compared to 2D or 3D scanning techniques.

Diffraction spot overlap: One of the principal limitations is related to the concept of orientation indexing based on a systematic search through scattering vectors, derived from diffraction spot peak positions. Acquisition conditions resulting in diffraction spot overlap will bring this concept to fail, because the erroneous determination of the centers of mass will result in erroneous determination of the associated scattering vectors and poor indexation results. The probability of diffraction spot overlap increases with the number of grains in the illuminated sample volume, the intragranular orientation spread, and the strength of texture. While it is difficult to provide absolute numbers for the individual conditions, due to the interplay of these different factors, it is possible to alleviate the probability of overlap by reducing the number of grains (by reducing the sample diameter and/or reducing the dimensions of the illuminated sample volume by closing the gap of the beam-defining slits), and optimizing the distance between sample and detector.

Number of grains: In the absence of deformation and texture, peak search based indexing techniques can work with up to a few thousand grains, while for a 5% deformed samples, a more realistic number is of order hundred.

Sample size: The sample dimensions in the direction perpendicular to the rotation axis should in general not exceed 20 grain diameters. Deformed and textures materials might require further reduction to values below 10 grain diameters.

Grain size: The minimum grain size limit is closely linked to the pixel size of the high resolution X-ray detector system employed. In order to spatially resolve 3D grain shapes, one should aim at about 10 voxels across the typical dimensions of the smallest grain size to be reconstructed. Given the physical limitation of high resolution X-ray detector systems to about $0.5\,\mu m$ resolution in the best case, the minimum grain size which can be handeled with type of approach is of order of 5-10 μm.

Scaling: Provided the material has a mono-modal grain size distribution, the pixel size can typically be adjusted over a wide range ($0.5\,\mu$m (in UO_2) up to $30\,\mu$m (in ice) have been demonstrated).

MATHEMATICAL DETAILS

Construction of the 6D reconstruction space

To work with high symmetry space-groups and limited values of intra-granular orientation spread, a regular sampling of Rodriguez parametrization of orientation space can be used (see [5] and suggestions therein for a computationally efficient representation applicable to all space groups, based on a compound description based on quaternions and "local" Rodriguez vectors). The voxel size in the real-space volumes will be defined by the acquisition resolution and the volume size can be estimated from the convex hull of the back-projected diffraction spots.

A natural choice of the orientation space resolution would be the angular step size used in the acquisition procedure. However, since an efficient implementation of the iterative reconstruction algorithm requires the full 6D volume to be loaded in computer memory, the sampling interval may have to be decreased to multiples of the acquisition step size in order to avoid saturation of the memory. Note that the orientation space resolution is linked to the total number of orientations and hence memory requirements by the third power of the inverse of the orientation step size. The use of adequate mathematical priors and interpola-

tion techniques discussed in [6] help to mitigate this problem and allow for the discretized orientations to represent the signal on the detector coming from the missing orientations. A different approach could be the reduction of the real-space resolution, to compensate for a finer orientation-space sampling grid, which could be used for reconstruction of bigger portions of the sample.

A conservative estimate of the bounding-box of the orientation distribution function of each grain can be obtained from the extreme values of the diffraction blob volumes in real space. The direction of the diffracted beam can be parametrized by two angles: 2θ being the (Bragg) angle between the diffracted beam and the incoming beam, and η being the angle between the projection of the rotation axis and the line connecting the intercepts of the direct beam (u_0, v_0) and the diffracted beam (u, v) on the detector (azimuth angle on the virtual Debye Scherrer ring associated to the current grain position).

While 2θ is not supposed to change in this 6-dimensional framework, for a given reflection, the deformation will be observed as a spread in both the ω and η directions. As described in [7], a given set of (θ, η, ω) angles will define a line in the Rodrigues representation of orientation-space. For a given reflection the lines produced by the extremal values of η and ω will not perfectly lie in the same plane, but the deviation will be small enough to use them to approximately define a limiting plane in orientation space for the Orientation Distribution Function of the grain being analysed. By collecting each of the four planes defined by the four extreme deviation values of η and ω for each reflection, it is possible to build a circumscribing polyhedron to the grain ODF.

Convex optimization algorithm

The Chambolle-Pock algorithm (CP, [8]) is a first-order primal-dual algorithm for convex optimization problems, which is among the most popular algorithms for solving total variation minimization problems. The CP algorithms can solve different types of optimization problems, and it is relatively easy to mathematically derive the algorithm 1, tailored for the formulation of the optimization problem in (eq. 3 of the article) [9].

A practical solution to avoid tuning of the free parameter λ in equation (3) of the article

consists in rewriting the functional as

$$\mathbf{x}^* = \underset{\mathbf{x}}{\operatorname{argmin}} || \left(|\nabla S \mathbf{x}| \right) ||_1 \tag{1}$$

$$\text{subject to: } \mathbf{x} \geq \mathbf{0} \text{ and } ||\boldsymbol{A}\mathbf{x} - \mathbf{b}||_2 \leq \epsilon \tag{2}$$

where ϵ is now an estimation of the total noise of the projection data. The formulation in equation 1 still contains a free parameter. However, performing a pre-selection of diffraction blobs based on intensity criteria one can reduce the noise level in the projection data. Considering the transition $\epsilon \to 0$ leads to:

$$\mathbf{x}^* = \underset{\mathbf{x}}{\operatorname{argmin}} || \left(|\nabla S \mathbf{x}| \right) ||_1 \tag{3}$$

$$\text{subject to: } \mathbf{x} \geq \mathbf{0} \text{ and } \boldsymbol{A}\mathbf{x} = \mathbf{b} \tag{4}$$

The CP algorithm instance tailored for this new form of the functional is given below in algorithm 2.

The parameter σ_1 is typically chosen as an approximation to the inverse of the l_2-norm of the matrix $\boldsymbol{A}$, while the parameter σ_2 is typically chosen as 1/2 which is the inverse of the l_2-norm of the matrix representation of the gradient.

If we consider that in our applications, the norm of the matrix $\boldsymbol{A}$ can be large (and therefore the parameter σ_1 is small), we see that $1 + \sigma_1 \approx 1$, such that Alg. 2 is almost identical to Alg. 1 with the choise $\lambda = 1$.

The algorithms (1) and (2) are made of few important pieces: a projection in both the projection data space and the space associated to the regularization condition in the objective function, the back-projection, and a memory step.

To understand how all these steps in the algorithm can be performed in our 6-dimensional problem (eq. 3 of the article) and (eq. 3), we will now introduce the underlying data-structures.

We have two main data-structures that hold the information: the collection of all the used diffraction blobs, also known as *diffstack* (vector $\mathbf{b}$) and the collection of all the volumes related to the sampled orientations, also known as *solution vector* (vector $\mathbf{x}$). This means that the *diffstack* contains all the information recorded by the detector, while the *solution vector* contains the 6-dimensional volume in orientation and real space, introduced in the

ALGORITHM 1: CP Instance for eq. 3 of the article

Require: x_0, y, λ

Ensure: Reconstruction in x_k

$\mathbf{p}_0 \leftarrow \mathbf{0}$

$\mathbf{q}_0 \leftarrow \mathbf{0}$

$\theta \leftarrow 1$

for $k \leftarrow 1, \ldots, n$ **do**

$\mathbf{p}_k \leftarrow \dfrac{\mathbf{p}_{k-1} + \sigma_1(\boldsymbol{A}\overline{\mathbf{x}}_{k-1} - \mathbf{y})}{1 + \sigma_1}$

$\mathbf{q}_k \leftarrow \lambda \dfrac{\mathbf{q}_{k-1} + \sigma_2 \nabla \boldsymbol{S} \overline{\mathbf{x}}_{k-1}}{max(\lambda \mathbf{1}, |\mathbf{q}_{k-1} + \sigma_2 \nabla \boldsymbol{S} \overline{\mathbf{x}}_{k-1}|)}$

$\mathbf{x}_k \leftarrow P_0\left(\mathbf{x}_{k-1} - \tau \boldsymbol{A}^T \mathbf{p}_k - \tau \boldsymbol{S}^T \operatorname{div} \mathbf{q}_k\right)$

$\overline{\mathbf{x}}_k \leftarrow \mathbf{x}_k + \theta\left(\mathbf{x}_k - \mathbf{x}_{k-1}\right)$

end for

ALGORITHM 2: CP Instance for eq. 3

Require: x_0, y

Ensure: Reconstruction in x_k

$\mathbf{p}_0 \leftarrow \mathbf{0}$

$\mathbf{q}_0 \leftarrow \mathbf{0}$

$\theta \leftarrow 1$

for $k \leftarrow 1, \ldots, n$ **do**

$\mathbf{p}_k \leftarrow \mathbf{p}_{k-1} + \sigma_1(\boldsymbol{A}\overline{\mathbf{x}}_{k-1} - \mathbf{y})$

$\mathbf{q}_k \leftarrow \dfrac{\mathbf{q}_{k-1} + \sigma_2 \nabla \boldsymbol{S} \overline{\mathbf{x}}_{k-1}}{max(\mathbf{1}, |\mathbf{q}_{k-1} + \sigma_2 \nabla \boldsymbol{S} \overline{\mathbf{x}}_{k-1}|)}$

$\mathbf{x}_k \leftarrow P_0\left(\mathbf{x}_{k-1} - \tau \boldsymbol{A}^T \mathbf{p}_k - \tau \boldsymbol{S}^T \operatorname{div} \mathbf{q}_k\right)$

$\overline{\mathbf{x}}_k \leftarrow \mathbf{x}_k + \theta\left(\mathbf{x}_k - \mathbf{x}_{k-1}\right)$

end for

"Method" section of the article. These two data structures are linked by a third object, generically named *geometry* (matrix $\boldsymbol{A}$). Each line of the matrix $\boldsymbol{A}$ contains the contribution of each element of $\mathbf{x}$ to one single pixel on the diffracted images, but using the ASTRA toolbox [10] it is possible to use a simpler description, which consists of a collection of tables that fully describe the projection of the 3D volumes onto the 2D detector images.

HARDWARE, SOFTWARE IMPLEMENTATION AND COMPUTATIONAL TIMES

The reconstructions were performed on the ESRF computing cluster and distributed over 10 machines with double Tesla K20 GPUs and 128GB of RAM. The software was implemented in Matlab[1] and C++, using the ASTRA Toolbox (*https://github.com/astra-toolbox*) for the projection and back-projection of the volumes. The computational times scale linearly with the number of sampled orientations, size of the real-space volumes and projection data, which means that for the smaller grains, reconstructions can take up to a few minutes, while for the biggest cluster reconstruction twelve hours are needed on one of the above-mentioned machines. The computation times could still be greatly reduced using the newer generations of graphics chipsets, because the biggest cost center in the reconstruction resides in the forward-projection and back-projection of the real-space volumes.

[1] Nikl, M. *et al.* Development of LuAG-based scintillator crystals - A review *Prog. Cryst. Growth Ch.* **59**, 47–92 (2013).

[2] Bay, B. K. Methods and applications of digital volume correlation. *J. Strain Anal. Eng.* **43**, 745–760 (2008).

[3] Gaye, A. *et al.* Micromechanics of Halite Investigated by 2D and 3D Multiscale Full-Field Measurements. *ARMA Conference Proceedings* (2014).

[4] Meyer, F. Energy storage in the grid. URL `http://www.bine.info/fileadmin/content/Publikationen/Projekt-Infos/2013/Projekt_18-2013/ProjektInfo_1813_engl_internetx.pdf` (2013) (*Date of access:* 22/12/2015)

[1] Registered trademark of *MathWorks*

[5] Schmidt, S. GrainSpotter : a fast and robust polycrystalline indexing algorithm. *J. Appl. Crystallogr.* **47**, 276–284 (2014).

[6] Viganò, N., Ludwig, W. & Batenburg, K. J. Reconstruction of local orientation in grains using a discrete representation of orientation space. *J. Appl. Crystallogr.* **47**, 1826–1840 (2014).

[7] Poulsen, H. F. *Three-Dimensional X-Ray Diffraction Microscopy*, vol. 205 of *Springer Tracts in Modern Physics* (Springer Berlin Heidelberg, Berlin, Heidelberg, 2004).

[8] Chambolle, A. & Pock, T. A First-Order Primal-Dual Algorithm for Convex Problems with Applications to Imaging. *J. Math. Imaging Vis.* **40**, 120–145 (2010).

[9] Sidky, E. Y., Jørgensen, J. H. & Pan, X. Convex optimization problem prototyping for image reconstruction in computed tomography with the Chambolle-Pock algorithm. *Phys. Med. Biol.* **57**, 3065–91 (2012).

[10] Palenstijn, W. J., Batenburg, K. J. & Sijbers, J. Performance improvements for iterative electron tomography reconstruction using graphics processing units (GPUs). *J. Struct. Biol.* **176**, 250–3 (2011). URL `https://github.com/astra-toolbox`.
